# Deletion of pH Regulator *pac-3* Affects Cellulase and Xylanase Activity during Sugarcane Bagasse Degradation by *Neurospora crassa*

**DOI:** 10.1371/journal.pone.0169796

**Published:** 2017-01-20

**Authors:** Amanda Cristina Campos Antoniêto, Wellington Ramos Pedersoli, Lílian dos Santos Castro, Rodrigo da Silva Santos, Aline Helena da Silva Cruz, Karoline Maria Vieira Nogueira, Rafael Silva-Rocha, Antonio Rossi, Roberto Nascimento Silva

**Affiliations:** 1 Department of Biochemistry and Immunology, Ribeirão Preto Medical School, University of São Paulo, Ribeirão Preto, São Paulo, Brazil; 2 Department of Genetics, Ribeirão Preto Medical School, University of São Paulo, Ribeirão Preto, São Paulo, Brazil; 3 Systems and Synthetic Biology Laboratory, Department of Cell and Molecular Biology, Ribeirão Preto Medical School, University of São Paulo, Ribeirão Preto, Brazil; University of California Riverside, UNITED STATES

## Abstract

Microorganisms play a vital role in bioethanol production whose usage as fuel energy is increasing worldwide. The filamentous fungus *Neurospora crassa* synthesize and secrete the major enzymes involved in plant cell wall deconstruction. The production of cellulases and hemicellulases is known to be affected by the environmental pH; however, the regulatory mechanisms of this process are still poorly understood. In this study, we investigated the role of the pH regulator PAC-3 in *N*. *crassa* during their growth on sugarcane bagasse at different pH conditions. Our data indicate that secretion of cellulolytic enzymes is reduced in the mutant Δ*pac-3* at alkaline pH, whereas xylanases are positively regulated by PAC-3 in acidic (pH 5.0), neutral (pH 7.0), and alkaline (pH 10.0) medium. Gene expression profiles, evaluated by real-time qPCR, revealed that genes encoding cellulases and hemicellulases are also subject to PAC-3 control. Moreover, deletion of *pac-3* affects the expression of transcription factor-encoding genes. Together, the results suggest that the regulation of holocellulase genes by PAC-3 can occur as directly as in indirect manner. Our study helps improve the understanding of holocellulolytic performance in response to PAC-3 and should thereby contribute to the better use of *N*. *crassa* in the biotechnology industry.

## Introduction

The filamentous fungus *Neurospora crassa* is an appealing microorganism for use by the bioethanol industry, because it degrades biomass releasing fermentable sugars [1]. Many isolates of this species were collected from sugarcane plantations [2–4] exhibiting the synthesis and secretion of holocellulolytic enzymes involved in plant cell wall degradation [5].

The *N*. *crassa* genome contains 171 genes encoding glycosylhydrolases, about 15.5% less than the cellulolytic model fungus *Trichoderma reesei*. However, the *N*. *crassa* genome possesses 35 genes encoding cellulolytic enzymes, almost twice that of *T*. *reesei* [6, 7]. Three types of enzymes are common to all cellulose-degrading fungi, including *N*. *crassa*: endoglucanases, exoglucanases, and β-glucosidases, which act synergistically to degrade the cellulolytic complex into glucose monomers [5].

The whole process of lignocellulosic biomass deconstruction is finely regulated at the transcriptional level. In *N*. *crassa*, the transcription factor XLR-1 (xylan degradation regulator 1) is necessary for growth on hemicellulose, whereas its orthologs, XlnR from *Aspergillus* sp. and XYR1 from *Trichoderma* sp., are the primary transcriptional regulators of genes encoding both cellulases and hemicellulases [8, 9]. Two other transcription factors, CLR-1 and CLR-2, also have an important role for the growth and induction of cellulases and some hemicellulases on cellulose; however, they are not required for growth on xylan [9, 10]. It is also known that the zinc-finger transcription factor CRE-1 is responsible for carbon catabolite repression (CCR) in *N*. *crassa* and that deletion of this regulator results in increased expression of cellulolytic genes and enhanced enzymatic activity during growth on cellulose [11].

In some species like *T*. *reesei*, *Aspergillus fumigatus*, and *N*. *crassa* [12–15], cellulase production is affected not only by available carbon source but also by the pH of the culture medium, indicating that pH is an important factor in the effective expression of these enzymes [12, 16]. The transcription factor responsible for the pH-signaling pathway is the protein PacC. In *Aspergillus nidulans*, this regulator mediates cell adaptation to pH by both activation of genes that are preferentially expressed in alkaline medium and repression of specific acid-associated genes [17]. At alkaline pH, PAC-3 is activated by PalB-catalyzed proteolysis, and active PAC-3 binds to the DNA of its target genes through the core consensus 5ʹ-BGCCVAGV-3ʹ in *N*. *crassa* [18].

Although a link has been demonstrated between PacC and lignocellulolytic enzyme production in species such as *A*. *nidulans*, *T*. *reesei*, and *Humicola grisea* var. *thermoideae* [12, 13, 19, 20], little is known about the role of PAC-3 in the expression of cellulases or hemicellulases in *N*. *crassa*. Here, we investigated the role of the transcription factor PAC-3 in the activity of holocellulolytic enzymes as well as in the regulation of cellulase and hemicellulase genes in *N*. *crassa*. We show that in alkaline environments, *pac-3* deletion results in decreased activity of endoglucanase and xylanase enzymes, as well as differential regulation of cellulase and hemicellulase gene expression. This study improves our understanding of the regulation of lignocellulosic biomass degradation in response to ambient pH in *N*. *crassa* for further application of metabolically engineered strains in the cellulosic production of ethanol.

## Materials and Methods

### Strains and growth conditions

Wild-type strain, St.L.74-OR23-1VA (FGSC No. 2489) and Δ*mus-52* (FGSC N°. 9568) (control) of *Neurospora crassa* were purchased from the Fungal Genetics Stock Center (FGSC; University of Missouri, MO, USA, http://www.fgsc.net) [21]. A *N*. *crassa* Δ*pac-3* knockout strain (*pac-3*^*KO*^) was generated following the knockout procedures described previously [22], based on the strain FGSC N° 9568 (mat a, mus-52::hyg) [23]. Strains were maintained on agar-based Vogel’s minimal (VM) medium at pH 5.8 [24], containing 2% sucrose at 30°C. For gene expression assays, conidia from each strain (approximately 10^7^ cells per mL) were germinated in 100 mL of liquid VM medium, supplemented with 1% (w/v) sugarcane bagasse (i.e., the carbon source) at 30°C in an orbital shaker (200 rpm) for eight days at different pH conditions (pH 3, 5, 7, 8, and 10). After this culture period, the resulting mycelia were collected by filtration, frozen, and stored at –80°C until RNA isolation. Supernatants were used for secretome and enzymatic activity analysis. Fungal growth was indirectly measured by total protein quantification using the Quick Start Bradford protein assay kit (Bio-Rad) with bovine serum albumin (BSA) as a standard. All experiments were performed in three biological replicates.

### RNA extraction and quantitative real-time analysis (RT-qPCR)

Total RNA was isolated from mycelia with the TRIzol RNA kit (Invitrogen Life Technologies, CA, USA), per the manufacturer's instructions. RNA concentration was determined by spectrophotometric OD 260/280, and RNA integrity was verified by gel electrophoresis in 1% agarose. For RT-qPCR analysis, 1 μg of RNA was treated with DNase I (Thermo Fisher, MA, USA) and reverse-transcribed to cDNA with the First Strand cDNA kit Maxima Synthesis, per manufacturer's instructions. The cDNA was diluted 1/50-fold and used for RT-qPCR analysis in the Bio-Rad CFX96^™^ System, using SsoFast^™^ EvaGreen^®^ Supermix (Bio-Rad, CA, USA) for signal detection. An actin-encoding gene (NCU04173) was used as an endogenous control. The following amplification reaction was used: 95°C for 10 minutes followed by 39 cycles of 95°C for 10 seconds and 60°C for 30 seconds, followed by a dissociation curve of 60°C to 95°C with an increment of 0.5°C for 10 seconds. Gene expression values were calculated according to the 2^-ΔΔCT^ method [25] and fold change expressed as Log_2_ using the 74A strain grown on sugarcane bagasse as the reference sample. Primers utilized in the RT-qPCR experiments are described in Table 1. Data analysis was performed using GraphPad Prism v 5.0 software. All samples were analyzed in triplicate.

**Table 1:** qRT-PCR primers used in this study.

GENES	Sequence 5′→3′ (Forward)	Sequence 5′→3′ (Reverse)
*cbh-1*	ACCCCCTCCACCAACG	GTGAACGCTGTAGAGACTTTGT
*gh7-1*	GCCCTACTCCAGCATTCAT	GTGAAGGCGGTGGCG
*gh3-2*	GGTGGAGAGGATGGATGAT	CCATCTGTTCGTTCGTCC
*gh11-1*	GGCTCTTACTCTGTGAACTGG	GTAGCCGTTGCCCGAG
*gh10-4*	GGGTGGGAGGAATGAAGA	ATCCTCGCCGCACACA
*gh43-5*	GAATGTTTATCAGGGCACG	GCTCATACCCAGTCCCG
*act*	ATGTCGACGTCAGGAAGGATCT	GAGCAGTGATCTCCTTCTGCAT
*clr-1*	GTTGTATTGTGCTTCTCGTCTTG	AACGCTGTTGGAGGTCATC
*clr-2*	ACGCAGTGTGGTTTCTCTAC	TCCAATACCGCCTCAATCTG
*cre-1*	GAGGATCACGATGACCACTATG	GAGTAGCAAGTGGAGTGTGATC
*xlr-1*	AGAGTACCACAGCAACCAAG	TTCGCTCATATCGCTCATGG

*cbh-1* = Cellobiohydrolase 1 (exoglucanase) NCU07340; *gh7-1* = Endoglucanase 1 NCU05057; *gh3-2* = β-glucosidase 1 NCU08054; *gh11-1* = Endo-1,4-β-xylanase 1 NCU02855; *gh10-4* = Endo-1,4-β-xylanase 2 NCU07130; *gh43-5* = β-xylosidase NCU09652. *clr-1* = Cellulose degradation regulator-1 NCU07705; *clr-2* = Cellulose degradation regulator-2 NCU08042; *cre-1* = Carbon catabolite regulation NCU08807; *xlr-1* = Xylan degradation regulator-1 NCU06971; *act* = actin NCU04173

### Enzymatic activities

Total cellulase activity (FPase) was determined by an enzymatic reaction employing Whatman filter paper no. 1, 30 μL of 100 mM citrate-phosphate buffer pH 5.0, and 30 μL of sample. The reaction was incubated at 50°C for 30 minutes. Next, 60 μL of dinitrosalicylic acid (DNS) was added to the reaction, which was then heated at 95°C for 5 minutes. Carboxymethylcellulase (CMCase) activity was determined following the protocol previously described [26] with some modifications. The reaction consisted of 30 μL of carboxymethylcellulose (CMC) prepared in 1% sodium acetate buffer (50 mM and pH 5.0) and 30 μL of sample. The reaction was incubated at 50°C for 30 minutes, followed by the addition of 60 μL of DNS, and an additional heating step at 95°C for 5 minutes. Xylanase activity was determined by reacting 50 μL of xylan substrate (xylan from beechwood [Sigma, MO, USA] at 50 mg/mL + 100 mM sodium acetate at pH 5.0) with 25 μL of sample at 50°C for 30 minutes. After incubation, 75 μL of DNS was added, and the reaction was heated at 95°C for 5 minutes. All enzymatic activities were performed in a 96-well microplate, and absorbance was read at 540 nm using the xMark^™^ Microplate Spectrophotometer (Bio-Rad, CA, USA). One enzyme unit was defined as the amount of enzyme capable of liberating 1 μmol of reducing sugar per minute [27].

### Western blotting

The proteins secreted by *N*. *crassa* were transferred to nitrocellulose (GE Healthcare, Buckinghamshire, UK). Rabbit secondary antibodies and anti-cellulase-*Trichoderma viride* antibodies (LS-C147688, LifeSpan Biosciences, WA, USA) were used as previously described [28]. Antibodies were visualized by enhanced chemiluminescence (Santa Cruz Biotechnology, TX, USA).

### In silico analysis of putative PAC-3 binding sites in *N*. *crassa* promoters

The complete genome sequence of *N*. *crassa* OR74A [29] was downloaded from https://www.broadinstitute.org/fungal-genome-initiative/neurospora-crassa-genome-project and was used to obtain the 5′ -upstream regions (1 kb) for each gene. The occurrence of the PAC-3 motifs 5′ -BGCCVAGV-3′ [30] were determined in the genes analyzed in this study, as well as for the 9730 genes in *N*. *crassa* genome, using an *ad hoc* Perl script. The motif frequency was calculated as the number of motif occurrences per kb.

### Statistical analysis

All tests of statistical significance were performed using one-way ANOVA (nonparametric) followed by the Bonferroni test (comparing all pairs of columns), available in the Prism software v. 5.0. Significance was indicated in the form of probability values (*P < 0.05, **P < 0.01, ***P < 0.001 and ****P < 0.0001).

## Results

### *pac-3* deletion affects cellulase and xylanase activities

**Fig 1:**
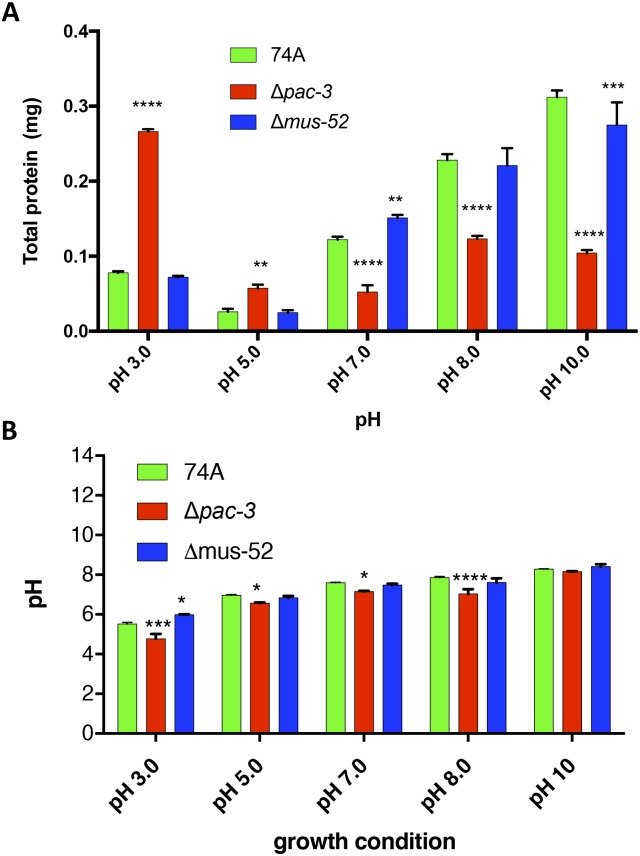
Total protein content (A) and pH changes (B) after growth of *N*. *crassa* for eight days on sugarcane bagasse. Strains 74A, Δ*mus-52*, and Δ*pac-3*, were cultivated on sugarcane bagasse at pH 3.0, 5.0, 7.0, 8.0, and 10.0 (initial pH). The final growth and pH was measured on the eighth day. The error bar indicates the standard deviation. *P < 0.05, **P < 0.01, ***P < 0.001, ****P < 0.0001.

Since the growth rate of the Δ*pac-3* mutant on solid medium is affected by pH [31], we investigated the final growth (total protein content) of 74A, Δ*pac-3*, and Δ*mus-52* in the cultured medium at pH 3.0, 5.0, 7.0, 8.0, and 10.0. The Δ*pac-3* mutant grew better at acidic (3.0 and 5.0) rather than neutral (7) or alkaline (8.0 and 10.0) pH when compared with 74A and Δ*mus-52* strains (Fig 1A). We conclude that the different growth rates observed in the Δ*pac-3* mutant can affect enzyme production; thus, all enzymatic activities were normalized by total protein content and expressed as specific activity. We also observed changes in the medium pH after the *N*. *crassa* strains 74A, Δ*pac-3*, and Δ*mus-52* were cultivated for eight days on sugarcane bagasse. However, only the Δ*pac-3* strain showed a significant difference at both pH 3 and 8, probably because of its poor growth under these culture conditions (Fig 1B).

**Fig 2:**
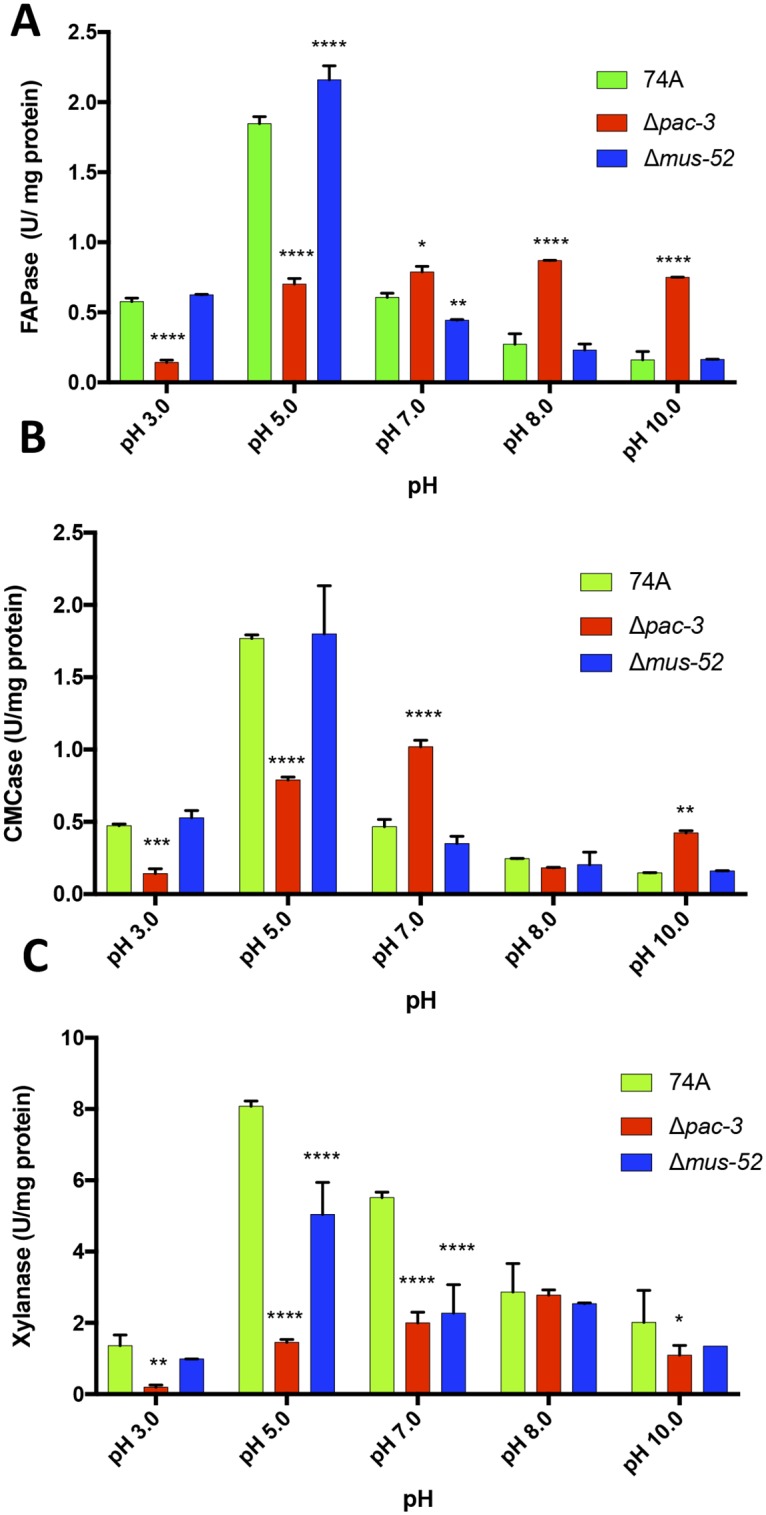
PAC-3 influences holocellulase activity in *N*. *crassa*. Total cellulolytic (FPase) (A), endoglucanase (CMCase) (B), and xylanolytic activities (C) were assayed in the supernatant of the strains 74A, Δ*mus-52*, and Δ*pac-3* after their cultivation for eight days on sugarcane bagasse. Values show the mean of three replicates. The error bar indicates the standard deviation. *P < 0.05, **P < 0.01, ***P < 0.001, ****P < 0.0001.

To determine whether PAC-3 affects the secretion of hollocellulolytic enzymes by the fungus, we analyzed the profile of total cellulolytic (FPase), endoglucanase (CMCase), and xylanolytic activities in strains 74A, Δ*pac-3*, and Δ*mus-52* (Fig 2).

With respect to total cellulolytic activity, we observed that the Δ*pac-3* strain showed nearly a 4-fold decrease in activity at an acidic pH of 3.0 and a 2.5-fold decrease at pH 5.0 as compared to that in the 74A strain (Fig 2A). However, after raising the pH to 7.0, 8.0, or 10.0, we observed an increase in specific FPase activity in the Δ*pac-3* strain (approximately 1-fold, 3-fold, and 4.5-fold, respectively) as compared to that in the parental strains, suggesting that the PAC-3 protein acts in neutral to alkaline pH by modulating the enzymatic activity and, consequently, the total specific cellulolytic activity (Fig 2A). As with FPAse activity, the same pattern was observed for the specific activity of endoglucanase (CMCase). At acidic pH environments, endoglucanase showed about 3-fold (pH 3.0) and 2.5-fold (pH 5.0) less activity than the parental strains (Fig 2B). At neutral pH, CMCase activity was approximately 2-fold higher than that of the parental strains. On the other hand, at alkaline pH, the endoglucanase activity only showed a significant difference at pH 10 (approximately 3-fold higher than the parental strains) (Fig 2B).

When the xylanase activity profile was evaluated, a significant decrease was observed in the mutant strain Δ*pac-3* as compared to the 74A and Δ*mus-52* strains at pH 3.0 (6.5-fold), pH 5.0 (5.5-fold), pH 7.0 (2.7-fold), and pH 10.0 (2-fold) (Fig 2C). Except at pH 8.0, the protein PAC-3 appears to upregulate the expression of xylanase activity regardless of the initial culture pH value (Fig 2C).

**Fig 3:**
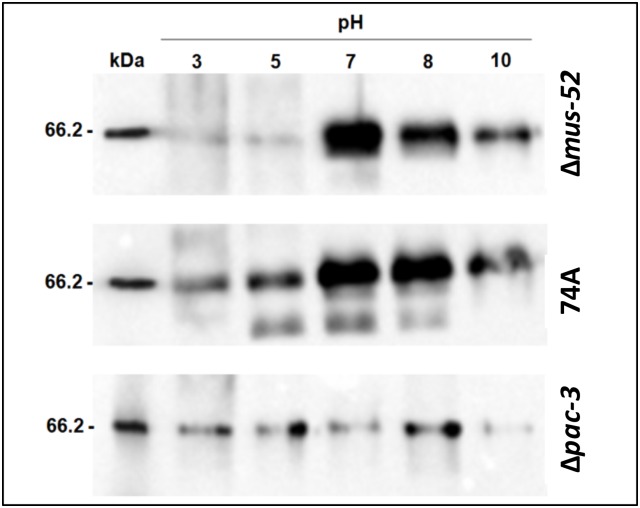
Western blot analysis shows a reduced amount of cellulase (CBHI) at both neutral and alkaline pH. The analysis was performed using supernatants collected from the *N*. *crassa* 74A, Δ*mus-52*, and Δ*pac-3* strains after growth for eight days on sugarcane bagasse at pH 3.0, 5.0, 7.0, 8.0, and 10.0. Anti-cellulase antibody from *Trichoderma viride* were used to detect CBH-1.

Since cellobiohydrolase I (CBH-1) is the most highly produced extracellular protein during *N*. *crassa* growth on cellulose [32], we performed western blot analysis to evaluate the influence of PAC-3 on cellulase secretion. Western blot analysis showed a high level of cellulase secretion in a *pac-3*^+^*mus*52^+^ background (strain 74A) irrespective of the initial pH but preferentially at neutral to alkaline pH (Fig 3). In a *pac-3*^+^*mus*52^−^ background (strain Δ*mus-52*), cellulase secretion preferentially occurred at neutral to alkaline pH. Unlike to observed for both strains, a decreased cellulase secretion was observed in the Δ*pac-3* strain at alkaline pH (pH 10.0). Thus, regardless of the initial culture pH, the protein PAC-3 affects the secretion of CBHI (Fig 3) as well as cellulase activity (Fig 2A).

### PAC-3 affects holocellulolytic genes in different ways

**Fig 4:**
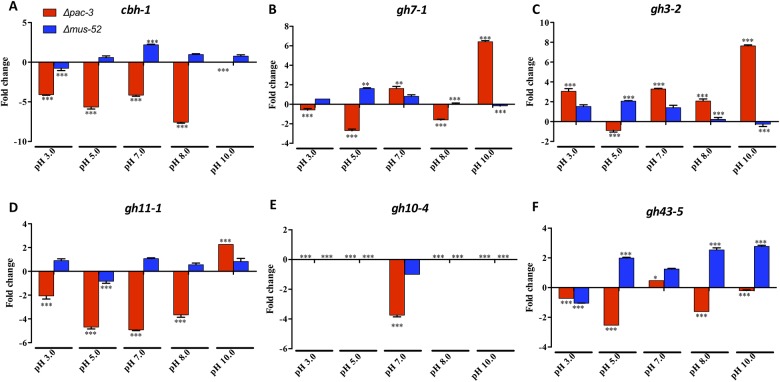
Gene expression profiles of holocellulolytic enzymes in *N*. *crassa*. The strains 74A, Δ*mus-52*, and Δ*pac-3* grown on sugarcane bagasse at different pH (3.0, 5.0, 7.0, 8.0, and 10.0) were used for RT-qPCR experiments. (A) *cbh-1* = Cellobiohydrolase 1 (exoglucanase) NCU07340; (B) *gh7-1* = Endoglucanase 1 NCU05057; (C) *gh3-2* = β-glucosidase 1 NCU08054; (D) *gh11-1* = Endo-1,4-β-xylanase 1 NCU02855; (E) *gh10-4* = Endo-1,4-β-xylanase 2 NCU07130; (F) *gh43-5* = β-xylosidase NCU09652. Values show the mean of three replicates. The error bar indicates the standard deviation. *P < 0.05, **P < 0.01, ***P < 0.001.

To evaluate the influence of the transcription factor PAC-3 on the expression of holocellulolytic genes, we used RT-qPCR to determine the expression profiles of the cellulases-encoding genes *cbh1*, *gh7-1* and *gh3-2* and the hemicellulases-ecoding genes *gh11-1*, *gh10-4* and *gh43-5* in the *N*. *crassa* strains 74A, Δ*pac-3*, and Δ*mus-52* (Fig 4).

The behavior of holocellulolytic genes was quite varied in response to deletion of the *pac-3* gene. Regarding cellulases, we observed that the absence of *pac-3* decreased the expression of *cbh1* in acidic (4-fold at pH 3.0 and 5.6-fold at pH 5.0), neutral (4-fold), and alkaline (7.5-fold at pH 8.0) media (Fig 4A). These results indicate that PAC-3 can serve as a positive regulator of *cbh1* in acidic, neutral, and alkaline media. Expression of the endoglucanase gene (*gh7-1*) in Δ*pac-3* revealed a reduction at pH 3.0 (0.5-fold), pH 5.0 (2.7-fold), and pH 8.0 (1.6-fold) and an upregulation at pH 7.0 (1.6-fold) and pH 10.0 (approximately 6.5-fold) (Fig 4B). In contrast, the expression of β-glucosidase 1 (*gh3-2*) increased as compared to the parental strains at all tested pH conditions—except at pH 5.0—reaching a maximum expression at pH 10.0 (approximately 7.5-fold increase) (Fig 4C). This result suggests that PAC-3 can act as a repressor of β-glucosidase during the growth of *N*. *crassa* on sugarcane bagasse, principally in alkaline medium.

Concerning the behavior of hemicellulases during the growth of *N*. *crassa* on sugarcane bagasse, we observed that expression of the *gh11-1* gene decreased at all tested pH conditions (reaching an almost 5-fold reduction at pH 7.0), except at pH 10 (approximately 2-fold higher) (Fig 4D). In contrast, expression of the *gh10-4* gene was null in the Δ*pac-3* strain in acidic and alkaline cultures (pH 3.0, pH 5.0, pH 8.0, and pH 10.0) as compared to that in 74A. However, at pH 7.0, the expression was dramatically reduced as compared to the parental strain (almost 4-fold) (Fig 4E). Thus, we believe that PAC-3 may be important in inducing the expression of the *ebx2* gene at neutral pH. Similar to the *gh11-1* gene, *pac-3* deletion caused a reduction of *gh43-5* expression in acidic and alkaline media (reaching 2.5-fold reduction at pH 5.0) (Fig 4F), suggesting that PAC-3 upregulates the expression of these genes.

Our data reveal that PAC-3 affects the expression of holocellulolytic genes and can serve as a positive or negative regulator, depending on the initial pH of the cultures. Therefore, this transcription factor may be involved in fungal adaptation to different pH conditions, implicating a critical role for PAC-3 in the regulation of lignocellulosic biomass deconstruction in *N*. *crassa*.

**Fig 5:**
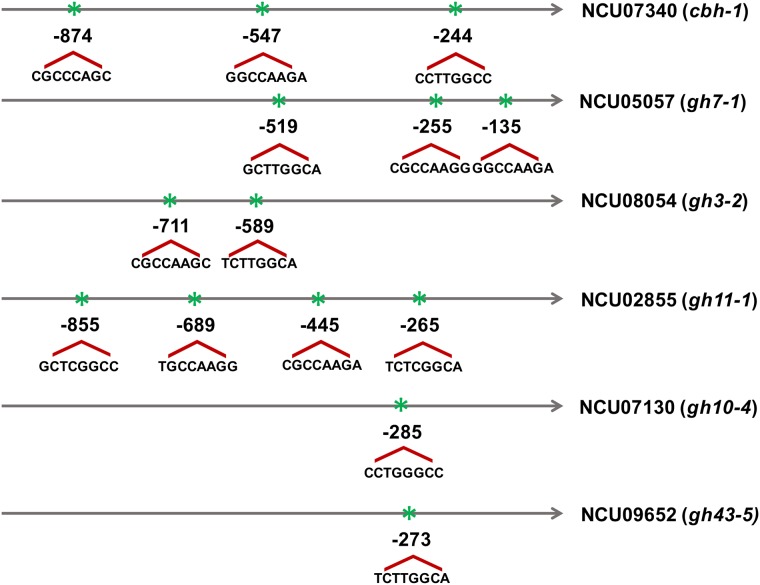
Schematic representation of putative PAC-3 binding sites (5′ -BGCCVAGV-3′). The analysis was performed using the region 1.0-kbp upstream of cellulolytic and xylanolytic genes. The position of the motifs is relative to the translation initiation codon (ATG). B = C or G or T; V = A or C or G, according to International Union of Pure and Applied Chemistry (IUPAC) norms.

In order to understand how PAC-3 regulates cellulase and xylanase gene expression, we performed a genome-wide analysis of PAC-3 binding motifs. The results are presented in Fig 5. As can be observed, all studied genes showed potential motifs for PAC-3 binding [18]. The cellulase genes *cbh-1* and *gh7-1* contained three potential sites, while the *gh3-2* glucosidase-encoding gene contained only two sites. For xylanases-encoding genes, the gene *gh11-1* showed four potential PAC-3 binding sites, whereas *gh10-4* and *gh43-5* only contained one site. This data suggests that the transcription factor PAC-3 can directly regulate the expression of cellulases and xylanases.

### The deletion of *pac-3* affects the expression of transcription factor-encoding genes

Although PAC-3 appears to directly regulate cellulase and xylanase genes, we decided to investigate if PAC-3 also influences the expression of the major transcription factors that are known to regulate hollocelluases genes [9, 11]: *xlr-1*, *cre-1*, *clr-1*, and *clr-2*. First, we performed genome-wide analysis of the PAC-3 binding motifs in the promoters (1 kb upstream the start codon) of genes encoding these transcription factors. Only the gene *cre-1* did not contain a potential binding site for PAC-3, whereas *xlr-1* contained four sites, followed by two sites for *clr-2*, and one site for *clr-1* (data not shown).

**Fig 6:**
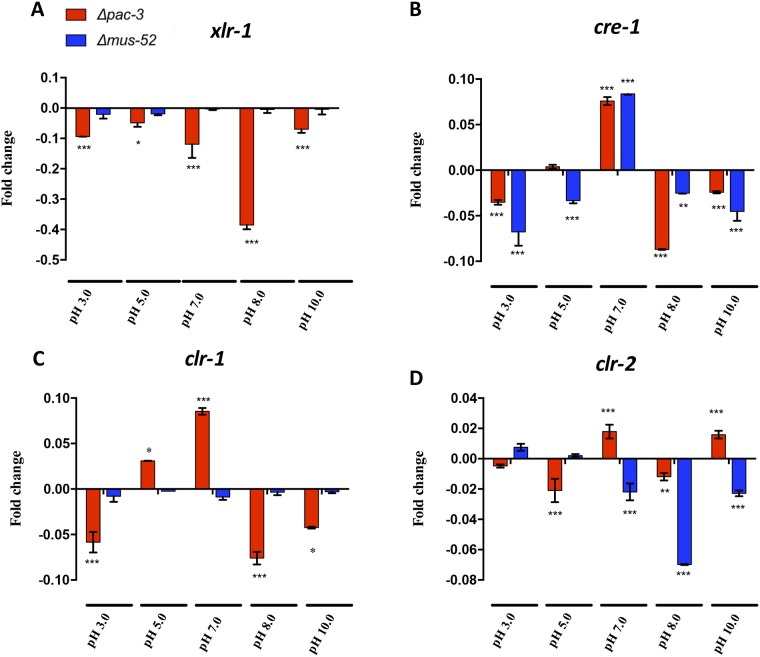
Effect of *pac-3* deletion on transcriptional factors gene expression. The strains 74A, Δ*mus-52*, and Δ*pac-3* grown on sugarcane bagasse at different pH (3.0, 5.0, 7.0, 8.0, and 10.0) were used for RT-qPCR experiments. (A) *xlr-1* = Xylan degradation regulator-1 NCU06971; (B) *cre-1* = Carbon catabolite regulator NCU08807; (C) *clr-1* = Cellulose degradation regulator-1 NCU07705 and (D) *clr-2* = Cellulose degradation regulator-2 NCU08042. Values show the mean of three replicates. The error bar indicates the standard deviation. *P < 0.05, **P < 0.01, ***P < 0.001.

The expression of transcription factor-encoding genes evaluated by RT-qPCR showed that, indeed, PAC-3 can regulate the expression of these factors, depending on pH conditions. Expression of *xlr-1* decreased in all tested pH conditions, showing a 0.5-fold reduction at pH 8.0 when compared to that in the parental 74A strain (Fig 6A). Although, *cre-1* does not contain a potential binding site for PAC-3, the expression of this gene was significantly decreased in acidic (pH 3.0) and alkaline (pH 8.0 and 10.) media, suggesting an indirect regulation of carbon catabolite repression mediated by CRE-1 by PAC-3 (Fig 6B). The expression of *clr-1* and *clr-2* were decreased in acidic (pH 3.0 and 5.0) and alkaline (pH 8.0) conditions. Notably, at pH 7.0, both transcription factors, *clr-1* and *clr-2*, were upregulated when compared to the strain 74A (Fig 6C and 6D). Also, we noted that the deletion of the gene *mus52* affected the expression of both *cre-1* and *clr-2* (Fig 6B and 6D). These results indicate that cellulase- and xylanase-encoding genes can be regulated by PAC-3 in both a direct and indirect manner.

## Discussion

Different pH conditions lead microorganisms to adapt physiologically to ensure their survival and reproduction. For this purpose, organisms have developed signal transduction pathways, allowing the secretion of enzymes with optimal activity at specific pH values. The regulation of gene expression and secretion of extracellular hydrolytic enzymes is part of this process and is crucial to improving lignocellulosic biomass degradation [33].

Our data show that the transcription factor PAC-3, a well-defined pH regulator in several species of filamentous fungi [12, 13, 19, 20], is also involved in the regulation of holocellulolytic enzyme production in *Neurospora crassa*. A study by Mello-de-Souza et al. [19] involving the thermophilic fungus *H*. *grisea* var. *thermoidea* showed that the transcription factor PacC is responsible for the regulation of those genes encoding cellulases and xylanases, which are strongly induced in sugarcane bagasse cultures at alkaline pH. This regulation can occur in a direct or an indirect manner. Another transcriptome study with the phytopathogen fungus *Colletotrichum gloeosporioides* showed that *pacC* deletion affects the expression of several genes, including cell wall-degrading enzymes [34]. Considering cellulase secretion, we observed that PAC-3 does not interfere with the expression of endoglucanase (CMCase activity) in acidic media, similar to that found by He and collaborators [12] in *T*. *reesei* during growth on cellulose. In this same study, they observed that total cellulolytic activity was higher in the mutant strain Δ*Trpac1* at neutral pH, as we showed for *N*. *crassa*, suggesting that *T*. *reesei* and *N*. *crassa* may share a similarity in PAC-3. However, the role of PAC1 in regulating cellulase-encoding genes in *T*. *reesei* is still unclear, since cellulase was not affected by *pac1* deletion in another study [20].

PAC-3 both positively and negatively regulates the expression of holocellulolytic genes during the cultivation of *N*. *crassa* on sugarcane bagasse. A study of *T*. *reesei* using genome-wide transcriptional analysis showed that, although *pac1* deletion affected several genes, few cellulases and hemicellulases were PAC1-regulated at pH 6.0, whereas β-glucosidase (Tr_47268) and alpha-1,6-mannanase (Tr_122495) were both downregulated in the Δ*pac1* mutant [20]. Analyzing the expression of a β-glucosidase (*bg1*), we noted that PAC-3 served as a repressor in both neutral and alkaline pH, different from that observed for the candidate β-glucosidase Tr_47268 from *T*. *reesei*. However, it is important to highlight that there are at least 11 β-glucosidases annotated in the *T*. *reesei* genome that could potentially be targeted for regulation. In contrast, our results agree with a previous report where the expression of *bgl1* increased dramatically in the mutant Δ*Trpac1* at neutral pH [12].

In *A*. *nidulans*, the induction or repression of cellulase genes is delayed in absence of the regulator PacC. RNA-sequencing analysis showed that genes encoding β-endoglucanases, cellobiohydrolases, and β-endoxylanases are among the major CAZy genes downregulated in Δ*pacC* mutant during growth on cellobiose at alkaline pH [13]. Our results also showed that in *N*. *crassa* there is a reduction in the expression of the genes *cbh-1*, *gh11-1*, and *gh10-4* in Δ*pac-3* compared to that of the parental strain at all analyzed pH conditions, except for *gh11-1* at pH 10.0. Also in *A*. *nidulans*, Perez-Gonzalez et al. [35] showed that during the growth on D-xylose, expression of β-xylosidase (*xlnD*) is partially reduced in the mutant strain *pacC*^*c*^14, suggesting that this transcription factor serves to positively regulate *xlnD*. Our results suggest a similar regulation in *N*. *crassa*, considering that the expression of the β-xylosidase gene *gh43-5* was also downregulated in the *N*. *crassa* Δ*pac-3* mutant.

A study involving the phytopathogen fungus *Alternaria alternata* showed that environmental pH has a large influence on the expression of the endoglucanase *AaK1* gene [36]. Examining the effect of PAC-3 on the expression of the *eg1* gene in *N*. *crassa*, we observed that the Δ*pac-3* mutant showed reduced expression of *eg1* at pH 8.0, whereas it increased considerably at pH 10.0. Thus, the effect of PAC-3 on the regulation of endoglucanase in *N*. *crassa* remains unclear.

Overall, the transcription factor PAC-3 is involved in secretion and gene expression regulation of cell wall-degrading enzymes. Notably, genes with similar functions can be positively or negatively regulated by PAC-3 depending on pH conditions, as found in *A*. *nidulans* [13]. This observation may be related to the dual regulatory role of PacC, previously described by MacCabe et al. [37], in which two xylanases from *A*. *nidulans* have opposite expression patterns depending on environmental pH. This interesting finding should promote more detailed studies in understanding how pH is sensed by *N*. *crassa* and how PAC-3 is involved in synergism of holocellulase action.

**Fig 7:**
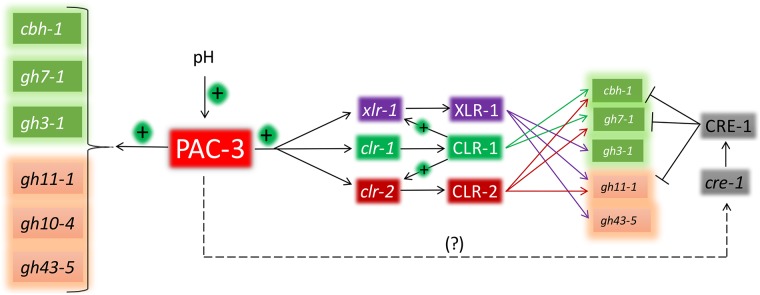
The model proposed for the regulation of holocellulolytic-encoding genes in *N*. *crassa* by PAC-3. Changes in the pH environment lead to activation of PAC-3, which directly or indirectly regulates transcription of cellulases and xylanases as well as the transcription factors XLR-1, CLR-1, and CLR-2 genes. The transcription factor CLR-1 regulate transcription of both *xlr-1* and *clr-2* genes. These transcription factors can also regulate cellulases and xylanases. XLR-1 regulate transcription of *gh3-1*, *gh11-1*, and *gh43-5* genes (purple arrows); CLR-1 regulate transcription of *cbh-1* and *gh7-1* genes (green arrows), and CLR-2 regulate transcription of *cbh-1*, *gh7-1*, and *gh11-1* genes (red arrows). (?) Missing component in regulation of *cre-1* by PAC-3.

In Summary, our results suggest that PAC-3 acts inducing total cellulolytic activity and endoglucanase activity at acidic pH environments whereas acting as a repressor of total cellulolytic and endoglucanase activity at neutral and alkaline pH. On the other hand, for xylanase activity, PAC-3 acts as a positive regulator in all pH environments (except at pH 8.0). It is well known that holocellulolytic enzymes are both directly and indirectly regulated [13, 19], by PacC. Our study showed that all studied genes can be directly targeted by PAC-3, probably at transcriptional level. On the other hand, PAC-3 can also regulate the major transcription factors that regulate the expression of holocellulase-encoding genes. Given the regulons of these transcription factors [9, 11] and our results, we propose a simple model of hollocellulase regulation by PAC-3 during sugarcane bagasse degradation (Fig 7).

Changes in the media pH activate the transcription factor PAC-3, which can then directly activate cellulase- and xylanases-encoding genes. In addition, PAC-3 can also directly activate *xlr-1*, which activates ß-glucosidase (*gh3-1*) as well as endo-xylanase (*gh11-1*) and ß-xylosidase (*gh43-5*). In this regulatory network, the transcription factor CLR-1 seems to have an important role. Directly, CLR-1 can activate only cellulases (*cbh-1* and *gh7-1*); however CLR-1 can also activate *xyr-1* and *clr-2* [9], which in turn will activate holocellulases-encoding genes. Finally, CLR-2 can activate both cellulases (*cbh-1* and *gh7-1*) and endo-xylanases (*gh11-1*) (Fig 7). CRE-1 mediates carbon catabolite repression, and PAC-3 can be involved in *cre-1* expression in an indirect manner. However, despite these evidences of regulation, how the overlapping regulation between PAC-3 and other transcription factors, especially CLR-1, is governed remains unclear. Indirect regulation or overlapping regulation with transcriptional factors seems to be related to the availability of low molecular weight inducers in *A*. *nidulans* [13]. However, more detailed studies will be necessary to elucidate the mechanism involved.

## Conclusion

Our data shows that the transcription factor PAC-3 from *N*. *crassa* influences the expression profile and secretion of cell wall-degrading enzymes during growth in sugarcane bagasse. At the different pH levels examined, we show that cellulase secretion is reduced in the absence of *pac-3* at an alkaline pH, and xylanolytic activity is positively regulated by PAC-3 at pH 5.0, 7.0, and 10.0. Regarding holocellulolytic genes, we observed differential regulation depending on the pH of the culture medium, suggesting that *N*. *crassa* can adapt to different environmental conditions. PAC-3 can also activate the major transcription factors that regulate holocellulase gene expression, suggesting that regulation occurs in both a direct and indirect manner. Ambient pH is a key factor in the industrial production of biomass-degrading enzymes. Our study contributes to a better understanding of the mechanisms involved in holocellulase production in response to pH, thereby improving its use in several industrial processes.
